# Gastric Pathology Image Classification Using Stepwise Fine-Tuning for Deep Neural Networks

**DOI:** 10.1155/2018/8961781

**Published:** 2018-06-21

**Authors:** Jia Qu, Nobuyuki Hiruta, Kensuke Terai, Hirokazu Nosato, Masahiro Murakawa, Hidenori Sakanashi

**Affiliations:** ^1^Department of Intelligent Interaction Technologies, University of Tsukuba, Tsukuba 305-8573, Japan; ^2^Department of Surgical Pathology, Toho University Sakura Medical Center, Sakura 285-8741, Japan; ^3^Artificial Intelligence Research Center, National Institute of Advanced Industrial Science and Technology (AIST), Tsukuba 305-8560, Japan

## Abstract

Deep learning using convolutional neural networks (CNNs) is a distinguished tool for many image classification tasks. Due to its outstanding robustness and generalization, it is also expected to play a key role to facilitate advanced computer-aided diagnosis (CAD) for pathology images. However, the shortage of well-annotated pathology image data for training deep neural networks has become a major issue at present because of the high-cost annotation upon pathologist's professional observation. Faced with this problem, transfer learning techniques are generally used to reinforcing the capacity of deep neural networks. In order to further boost the performance of the state-of-the-art deep neural networks and alleviate insufficiency of well-annotated data, this paper presents a novel stepwise fine-tuning-based deep learning scheme for gastric pathology image classification and establishes a new type of target-correlative intermediate datasets. Our proposed scheme is deemed capable of making the deep neural network imitating the pathologist's perception manner and of acquiring pathology-related knowledge in advance, but with very limited extra cost in data annotation. The experiments are conducted with both well-annotated gastric pathology data and the proposed target-correlative intermediate data on several state-of-the-art deep neural networks. The results congruously demonstrate the feasibility and superiority of our proposed scheme for boosting the classification performance.

## 1. Introduction

Cancer is acknowledged as one of the top threats to human health. According to the International Agency for Research on Cancer (IARC), in 2012, there were approximately 14.1 million new cancer cases and 8.2 million deaths around the world [1]. This number is estimated to increase to 24 million by 2035, and the deaths will continually rise. Within the diagnostic methods, although advanced image diagnosis devices (computed tomography (CT), magnetic resonance imaging (MRI), ultrasound (US)) are evolving rapidly, for many kinds of cancers, pathological diagnosis is still realized as the gold standard to assess cancer's presence or absence, type, and malignance degree. However, the shortage of pathologists represents as great restriction to pathological diagnosis and causes social problems. In the United States, the lack of pathologist workforce is become more and more concerned [2]. In Japan, the number of pathologist normalized by the general population is even smaller than one-third of that in the United States (one pathologist per 19,000 people) [3]. The situation is even more severe in China. As reported, China has approximately one pathologist per 74,000 people [4]. Such severe shortage is now consequently leading to immense working burden on pathologists and possible errors and oversights in diagnosis. Compared with 2005, the number of all pathological diagnosis cases and intraoperative pathological diagnosis cases in Japan has, respectively, risen up to 1.7 times and 3 times by 2012. New technologies such as digital pathology has widely spread and facilitated faster and cheaper diagnosis since more than a decade ago, due to its operational ease enabled by virtual microscopy [5, 6]. Nevertheless, since diagnosis correctness and pathologist workload alleviation remain challenges, further assistance based on advanced image classification technologies is expected to play a key role to facilitate more advanced computer-aided pathology diagnosis.

In earlier periods, conventional classification methods for pathology images including specified histologically concerned features or generalized texture image features are commonly adopted. The specific histologically concerned features, such as nuclei's area and nuclear-cytoplasmic ratio (N/C), are subtly calculated from unknown images [7]. These features are compared with predefined criteria to judge whether the target image is benign or malignant. Unfortunately, such process usually meets a big issue that it is a hard task to make adequate definition for the morphological characteristics, because cancerous cells usually lack control for regular division. Thus, shape extraction failures for cells could become a direct reason for classification failures. In contrast, the schemes using generalized texture image features appear to sustain more robustness to various cancerous appearances. As a customary way, many of the previous studies taking advantage of generalized texture feature have demonstrated their capability for respective tasks. One of the focused texture feature is grey-level co-occurrence matrix (GLCM). For example, Esgiar et al. [8] employed GLCM to obtain texture features corresponding to contrast, entropy, angular second moment, dissimilarity, and correlation from colon biopsy and employed linear discriminate analysis (LDA) and k-nearest neighbour algorithm (KNN) to realize the categorization of normal and cancerous colon mucosa. Likely, Diamond et al. [9] employed Haralick features (a kind of texture features developed from GLCM) for identifying tissue abnormalities in prostate pathology images. Another mighty rival is local binary patterns (LBPs). In the study of Masood and Rajpoot [10], a scheme consisting of LBP and support vector machines (SVMs) is proposed and demonstrated effective for colon pathology images. In another work, Sertel et al. [11] developed classification methods for neuroblastoma H&E-stained whole-slide images, using co-occurrence statistics and local binary patterns similar to the above study. A recent report by Kather et al. [12] gave a relatively comprehensive investigation of texture analysis for colorectal cancer histology image. Besides LBP and GLCM, lower-order and higher-order histogram features, Gabor filters, and perception-like features are involved as well. In our earlier studies [13], another texture features called higher-order local autocorrelation (HLAC) bonded with linear statistical models such as principal component analysis (PCA)-based subspace method were also demonstrated capable of indicating the anomaly degree of gastric pathology images. Apart from straightforward benign/malignant classification, some other methods in pathology image domain have been put forward with texture features as well, to settle similar classification-correlative tasks such as gland segmentation and grade estimation [14–19].

While all of these texture-feature-based approaches shown promising feasibility, intractable issues still existed between the research and practical application. One particular instance is that confirming how suitable the hand-crafted geometric features are for certain tasks is quite difficult [20]. Meanwhile, the uneven H&E staining among images brings adverse impact on classification performance so that it makes the tasks more challenging [21–23]. Since the dominative victory of the team using deep learning at ImageNet Large Scale Visual Recognition Competition (ILSVRC) 2012, many of the image recognition techniques have been replaced by deep learning using convolutional neural networks (CNNs) [24]. Due to more domain agnostic approach combining both feature discovery and implementation to maximally discriminate between the classes of interest [25], deep learning shows unprecedented adaptability for various kinds of images [26–28]. Accordingly, high hope is placed on deep learning to exert great power in pathology image and other medical image analysis fields [29–33]. Specifically within the pathology image domain, in addition to aspiration for more precise classification and segmentation [22, 34–38], deep learning has also been inspired for new patulous applications, such as stain normalization [39], assessment of tumor proliferation [40], and comprehensive multimodal mapping between medical images and diagnostic reports [30].

In comparison with computer vision for natural images, scarcity of training data along with accurate annotations in the medical image field has currently become a primary issue. Due to the necessity of medical doctor's knowledge and collaboration, acquisition procedures usually cost both expensive financial resources and workload. In consideration to this problem, many of the studies have evidenced that transfer learning using fine-tuning techniques for deep neural networks can boost the performance and alleviate the scarcity of training data in some degree by transferring a general neural network pretrained by large-scale image datasets (such as ImageNet), to a more specified one corresponding to more complicated target tasks [22, 41–43]. However, since pretraining image datasets possess fixed categories of contents and image size, the coefficient efficiency of the datasets and deep neural network is generally suboptimal for problems encountered in specific image classification domain [44]. In many cases, there are still gaps between the knowledge gained from pretrained tasks and different specified target domains.

Hence, in this paper, aiming to further alleviate the scarcity of well-annotated training data for gastric pathology image classification and enhance the performance in a rational way, we have proposed a novel scheme adopting two-stage fine-tuning approach for CNNs and introduced a new type of target-correlative intermediate datasets (called “medium-level” datasets, hereinafter). In addition to the “low-level” large-scale pretraining datasets owning enormous amount but little target specificity, and the “high-level” well-annotated pathology datasets directly related to the target task, the proposed “medium-level” datasets are produced based on tissue-wise and cell-wise information within pathology image domain. With the “medium-level” datasets, our scheme is supposed capable of making the deep neural networks imitating the perception manner of pathologists and acquiring pathology-related knowledge in advance, but with very limited extra cost in data annotation. In the following parts, we will give the detailed materials of both the stepwise fine-tuning scheme and the datasets. After that, experiments will be organized to evidence our proposed scheme.

## 2. Materials and Methods

**Figure 1:**
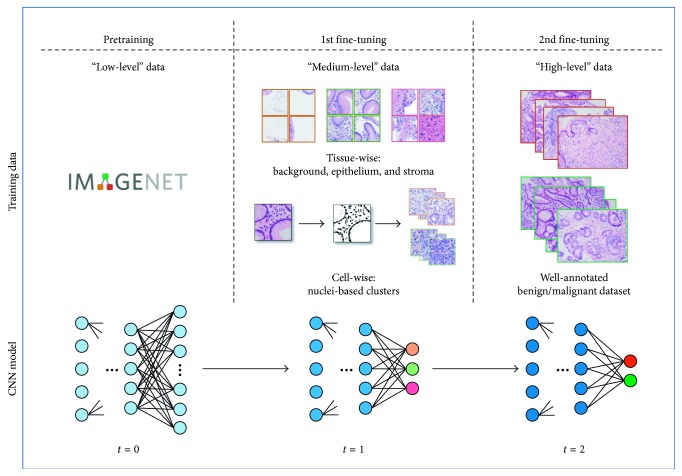
The proposed stepwise fine-tuning architecture. Apart from “low-level” pretraining datasets and well-annotated “high-level” datasets, “medium-level” data including tissue-wise data and cell-wise data are involved as well. In CNN models corresponding to training step *t* = 0, *t* = 1, and *t* = 2, blue nodes coloured darker denote more specified (deeper) representation for the pathology image classification task.

This section will be unfolded to several subtopics. Firstly, we will make a brief instruction of convolutional neural networks (CNNs) and the architectures adopted in this paper. Secondly, we will give some details about the proposed stepwise fine-tuning scheme (Figure 1) to clarify how it helps to improve CNN's performance and alleviate the insufficiency of well-annotated pathology training data. Thirdly, we will represent a feasible tissue-wise “medium-level” dataset including classes of background, epithelium, and stroma, which can be semiprofessionally annotated based on fundamental pathological knowledge with little pathologist's workload. Lastly, supposing to minimize the annotation cost and give an alternative solution to extremely insufficient annotating workforce, we are further inspired to put forward a solution by implementing full-automatic production of the cell-wise “medium-level” datasets which are yielded upon nuclei measurement and unsupervised clustering.

### 2.1. Convolutional Neural Network (CNN) Architecture

Among various approaches which have been studied in the field of image recognition and computer vision, convolutional neural network (CNN) is currently the most remarkable success. The prototype of CNN can be found in neocognitron [45] devised based on the neurophysiological findings on the visual cortex of living organism's brain. It is a neural network that alternately arranges a convolution layer corresponding to the cells for feature extraction and a pooling layer corresponding to the cells having a function to allow positional deviation hierarchically. Intuitively, it can be interpreted as a network that takes co-occurrence of adjacent features on different scales little by little and selectively gives information effective for identification to upper layers. Practically, refinement of such information is usually implemented by minimizing the cost function:

(1) $$\begin{array}{c} L\left(W\right)=\frac{1}{N}\sum_{i=1}^{N}l\left(y\left(x_{i};W\right),y_{i}\right). \end{array}$$

In (1), while *L*(*W*) indicates the total cost (difference between prediction upon current configuration and the ground truth) over a dataset of *N* training samples, corresponding to weights *W*. *y*_*i*_ denotes the label of training data *x*_*i*_. *y*(*x*_*i*_; *W*) is the predicted label of *x*_*i*_, while *l* is the lost function. Practically, in order to evaluate the efficacy and generalization of the proposed stepwise fine-tuning scheme, in this paper, we employ several state-of-the-art CNN architectures including VGG-16 [28], AlexNet [24], and GoogLeNet (Inception V3, hereinafter) [46].

### 2.2. Making CNN Learn Pathology by Stepwise Fine-Tuning

The fine-tuning approach, which generally indicates retraining the pretrained CNN with the dataset corresponding to the target task, is widely adopted in image classification domain [47–52]. Since training a CNN strongly depends on initial parameters, it is significant to obtain appropriate parameter initialization as much as possible in order to prevent overfitted learning. Generally, the early layers within a CNN are in charge of acquiring relatively universal image features, which are considered analogous to the conventional texture features and applicable to many of related tasks, while the later layers are involving more specific information corresponding to the target task. Therefore, if the distributional difference between the datasets for pretraining and target is sufficiently correlated, one may fine-tune part of or all the layers to yield more desired results than those train from full scratch in many cases. However, in other situations if target tasks possess much different distribution compared with the pretraining datasets, effectiveness of initialization and fine-tuning may be largely restricted. Such issue is exactly posed in pathology image classification. In light of human's perception, pathology images usually have more complicated appearances than natural images (included in the pretraining dataset) since it is difficult to figure out the intuitionistic difference between benign and malignant images due to their color uniformity by H&E stain. From this perspective, we believe that it is necessary to make some substantial effort to fill up the “gap” between the tasks of pretraining dataset and well-annotated pathology dataset.

Therefore, in this paper, a novel conception is proposed if it is possible to make deep neural networks learn to understand pathology images in pathologist's way. With regard to the perception manner and learning progress of a pathologist, before drawing conclusions of benign or malignant for a pathology image, the pathologist should understand the difference of basic tissue-wise structures beforehand. Then, the pathologist may concentrate more on the detailed morphological characteristics, including but not limited to the spreading status and density of cells, degree of nucleus distortion, nucleus size, and nuclear-cytoplasmic ratio. On account of all of the above observations, the pathologist can finally make a benign or malignant judgement. To realize such conception, we build up a stepwise fine-tuning scheme to impart the intellectual pathology knowledge on deep neural networks, and introduce a new type of target-correlative “medium-level” dataset. Rather than directly driving the deep neural networks to learn how to identify benign or malignant, making it gain fundamental pathological knowledge from the “medium-level” datasets probably contributes to the task of higher difficulty. Therefore, based upon the in-being framework, training with the “medium-level” datasets is placed in the middle of the stepwise fine-tuning scheme [53]. As shown in Figure 1, at the beginning of the training progress *t* = 0, we have a pretrained network as initialization. The following step *t* = 1 is fine-tuning the network with our proposed “medium-level” dataset. After this step, the deep neural network is considered to possess more pathology-related weights. Finally, well-annotated benign/malignant images are used for the second time (*t* = 2) fine-tuning. In the lower part of the figure, corresponding to training step *t* = 0, *t* = 1, and *t* = 2, blue nodes coloured darker in CNN models denote more specified (deeper) representation for the pathology image classification task. When the number of output classes changes, definitely, the network architecture needs to be adjusted accordingly.

### 2.3. Building Up a Low-Cost “Medium-Level” Dataset

**Figure 2:**
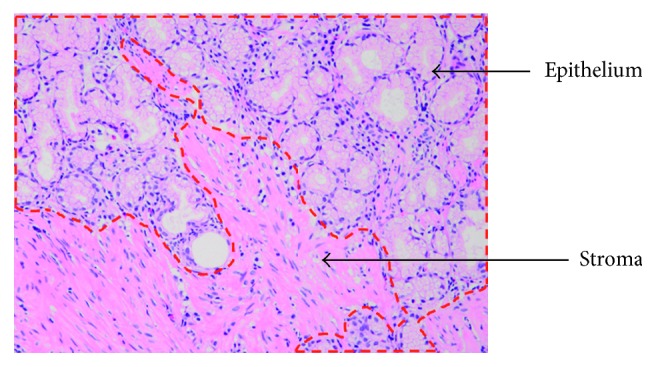
Stroma tissue and epithelium tissue appear in gastric pathology image.

As for the “medium-level” datasets, it is supposed necessary to meeting the prerequisite requirement that they could be acquired at much lower cost than the datasets annotated by pathologists and meanwhile charged with fundamental pathology related knowledge. Therefore, we firstly pay attention to seeking for a kind of pathology image datasets which can be made by “nonprofessional” workforce. In this paper, we present a feasible stroma-epithelium datasets which can be made by ourselves with only a little pathologist's direction. Epithelium and stroma are two tissue types that can be found in every organ [54]. In gastric pathology domain, epithelial tissues line the outer surfaces of gastric mucosa, while stroma tissue locates right under epithelium. Since cancer metastasis between epithelium and stroma is deemed as inextricable to cancer's progression [55], both of the two types of tissue are usually extracted during biopsy examinations and revealed in the pathology images. Although it is quite difficult to identify if the two types of tissue are in order for nonprofessional workers, actually, we find that stating the visual difference between them is quite an undemanding work. As shown in Figure 2, the upper area encircled by dashed contour indicates epithelium tissue, while the remaining lower area denotes stroma tissue. Because of their respective functions, obviously, epithelium tissue appears as organized arrangement, but stroma tissue seems scattered and disordered. Such distinct difference is considered quite beneficial to our “nonprofessional” work. Without pathologist's expensive annotation, it would be encouraging if these epithelium and stroma images can impart the pathology knowledge to the deep neural networks on the basis of our assumption. Practically, we have managed to collect a large amount of images including epithelium and stroma, segment them with stroma area and epithelium and manually, and separate into three classes (epithelium, stroma, and background).

### 2.4. Automatically Generating a “Medium-Level” Dataset

In this section, this paper moves one step further and presents another full-automatic “medium-level” dataset generating approach, corresponding to the situation when data annotation workforce is extremely insufficient. The proposed approach firstly applies a string of image processing techniques, before extracting low-dimension feature vectors indexing the measurement of morphological status of nuclei. Finally, we perform unsupervised clustering in order to form the “medium-level” dataset.

In order to realize the measurement of nuclei, it is necessary to generate a binary nuclei image instead of the original one. Thus, the first step is to implement color deconvolution to separate H&E- (hematoxylin and eosin-) stained pathology image into H and E component, since nuclei are usually richly visualized in H component. This paper adopts the algorithm proposed by Ruifrok and Johnston [56]. According to their work, although relative intensity in each of RGB channels depends on the concentration of stain in a nonlinear way, the optical density, *OD*_*c*_=−log10(*I*_*c*_/*I*_0_, *c*)=*A∗b*_*c*_ (*c* is any of the RGB channels), is linear with the concentration of amount of stain *A* with absorption factor *b*_*c*_ and therefore can be used for separation of the contribution of multiple stains. Practically, *I*_*c*_ is the intensity of one of RGB channels and *I*_0_, *c* is valued 1. Then, with the deconvolution matrix *D* denoted as (2), combination of hematoxylin, eosin, and DAB can be calculated as *C*=*D*[*y*], where [*y*] indicates vector of optical density in RGB space:

(2) $$\begin{array}{c} D=\left[\begin{array}{ccc} 1.88 & -0.07 & -0.60\\ -1.02 & 1.13 & -0.48\\ -0.55 & -0.13 & 1.57 \end{array}\right]. \end{array}$$

Next, we perform binarization on H (hematoxylin) channel, with the auto local threshold algorithm presented by Phansalkar et al. [57]. In this method, the threshold *T* is computed as

(3) $$\begin{array}{c} T=\mu^{*}\left(1+p^{*}e^{-q*\mu }+k^{*}\left(\left(\frac{\sigma }{r}\right)-1\right)\right). \end{array}$$

**Figure 3:**
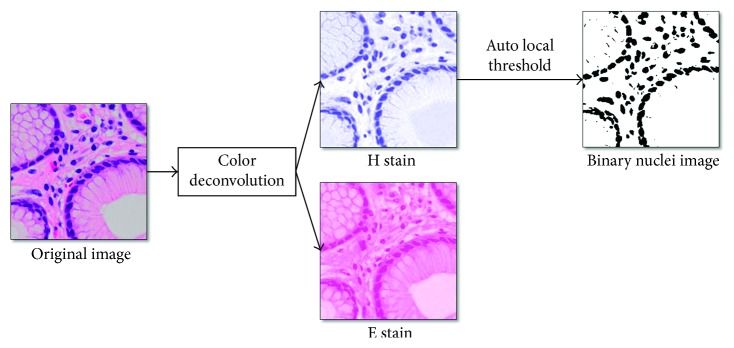
Image processing for making binary nuclei images.

In (3), *μ* and *σ* are the local mean and standard deviation, respectively. *k* = 0.25, *r* = 0.5, *p* = 2, and *q* = 10 are constants with their recommended values. The image on the right in Figure 3 shows the result after banalization.

Afterwards, our proposed method adopted watershed algorithm for separating the conjoined or partly overlapped nuclei and then the contour detection method was employed, developed by Suzuki and Abe [58], in order to acquire a two-dimensional vector of total area and number of nuclei from each pathology image. After all, unsupervised K-means clustering is utilized to separate images into two classes. The clustered images are directly used as “medium-level” training data in the first time fine-tuning. Compared with tissue-wise dataset, the automatically generated dataset concentrates on cell-wise characteristics.

## 3. Results and Discussion

### 3.1. Experimental Procedures

In order to evaluate the effectiveness of our proposed stepwise fine-tuning scheme and the low-cost “medium-level” datasets, the experiments are progressively arranged in the following order. (1) We firstly give discussion on the performances of the two-stage fine-tuning scheme based on different CNN architectures when tissue-wise dataset is employed. (2) We then replace the tissue-wise dataset with automatically generated cell-wise dataset and validate the proposed scheme in the same way as (1). Separately in (1) and (2), we will further talk over how our proposed two-stage scheme performs upon well-annotated datasets in different sizes, in order to produce more evidence to support our conception.

### 3.2. Datasets

**Figure 4:**
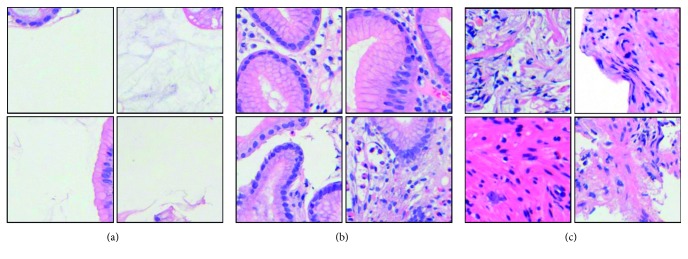
“Nonprofessionally” annotated tissue-wise datasets. (a) Background. (b) Epithelium. (c) Stroma.

**Figure 5:**
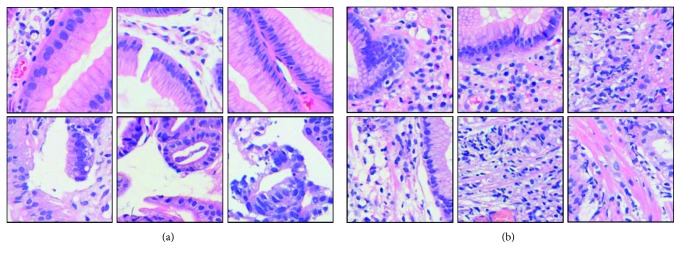
Full-automatically generated cell-wise (nuclei) datasets. (a) Cluster 1. (b) Cluster 2.

This paper employs three types of data including “low-level,” “medium-level,” and “high-level” data (Figure 1), respectively, used for the initialization, the first-stage fine-tuning, and the second-stage fine-tuning. In practice, ImageNet data [59] containing approximately 1.2 million images in 1,000 separate categories are customary utilized to initialize the CNN models. As to gastric pathology images, all the datasets utilized are illustrated in Table 1. By depicting maps of epithelium and stroma, we succeeded in collecting 48,000 tissue-wise patches (256 × 256) separated into “background,” “epithelium,” and “stroma” categories (Figure 4). In each category, 15,000 patches are used as training data, while the remained 1,000 patches are used for validation. From this dataset, we further obtained automatically generated cell-wise dataset in line with nuclei measurement. The cell-wise dataset consists of 7,672 and 6,457 patches in two clusters (Figure 5), after excluded those having too few nuclei or difficult to be extracted due to stain inconformity. Experimentally, 90% of patches out of each cluster are used for training and the rest are used for validation. As shown in Figure 5, according to rough observation, the cluster on the left appears to have larger and fewer nuclei, while the cluster on the right side seems opposite with more but smaller nuclei. As to the well-annotated “high-level” datasets, in order to evaluate the efficacy and generalization of the proposed two-stage scheme, we have prepared well-annotated datasets in two different sizes. One is a small train dataset including 540 benign and 540 malignant patches. Another one is a nonaugmented large dataset of 5,400 benign and 5,400 malignant patches within which the small dataset is included. Except from the former datasets, we additionally use a validation dataset including 1,620 benign and 1,620 malignant patches to select the best model configuration, and a test dataset of 2,700 benign and 2,700 malignant patches to finally evaluate the performance in each optional case. It is noteworthy that there is no overlap between the “medium-level” datasets and the “high-level” datasets and no overlap among the training, validation, and test datasets.

## 4. Experimental Results

### 4.1. Stepwise Fine-Tuning Using Tissue-Wise Data

In this part, we will specifically discuss about our proposed stepwise fine-tuning when tissue-wise data are employed for the first-stage fine-tuning and well-annotated datasets are used for the second-stage fine-tuning. The performances are collected from the experiments performed with well-annotated pathology image datasets in different sizes and different deep neural network architectures. In this paper, we concurrently adopt AUC, ACC, precision, and recall as the evaluation criteria [60].

**Figure 6:**
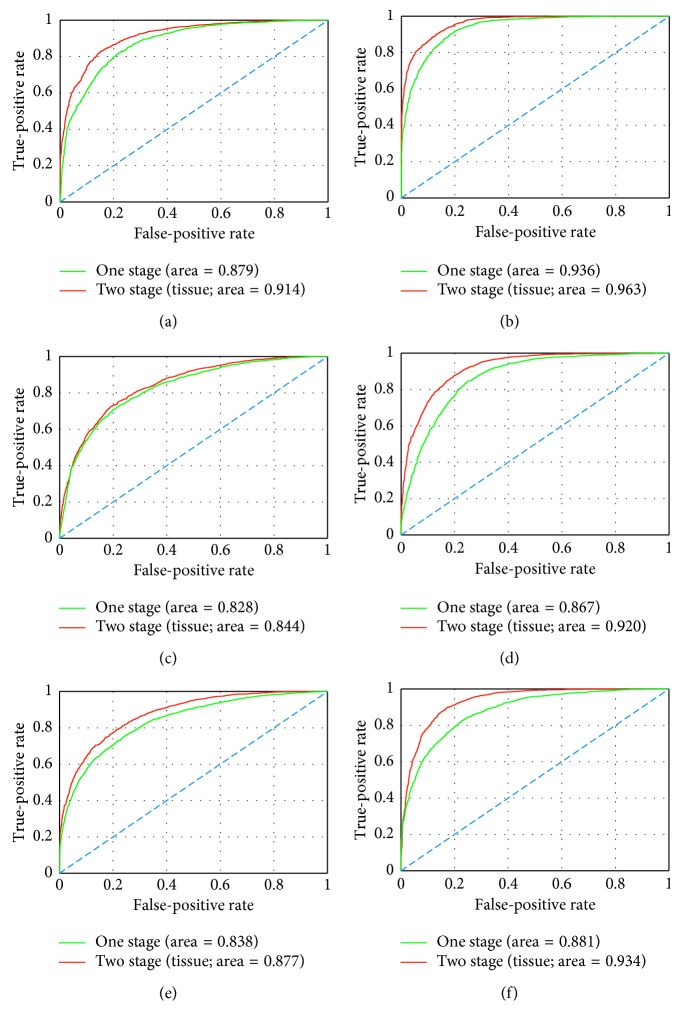
Performances of the proposed two-stage fine-tuning using tissue-wise data presented by ROC. (a) VGG-16 + small data. (b) VGG-16 + big data. (c) AlexNet + small data. (d) AlexNet + big data. (e) Inception V3 + small data. (f) Inception V3 + big data.

**Figure 7:**
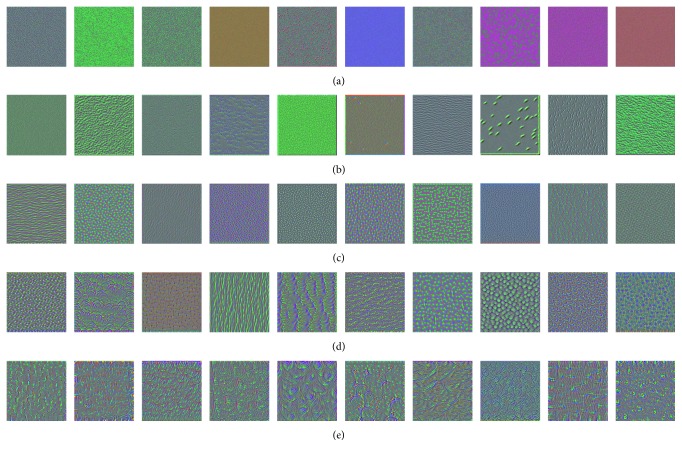
A set of filtered response images outputted by the stepwise trained VGG-16 model. (a) Block 1, Convl. (b) Block 2, Convl. (c) Block 3, Convl. (d) Block 4, Convl. (e) Block 5, Convl.

In Table 2, notably, in all of the couples of rival schemes, our proposed two-stage fine-tuning using tissue-wise data has yield promotion. Specifically, in the results of small data group, AUC value is raised by 0.035, 0.016, and 0.039, when we adopt CNN architectures VGG-16, AlexNet, and Inception V3, respectively. In the large data group, the corresponding AUC values are raised by 0.027, 0.053, and 0.053. Although the performance using smaller training data is expected to be more boosted, according to AUC values, we find that our proposed scheme has actually brought slightly more benefit to the large well-annotated dataset groups. Meanwhile, if we focus on ACC values, we are aware of the fact that the greatest improvement happens when our proposed scheme using Inception V3 is adopted upon the large dataset. The accuracy has remarkably increased from 0.779 to 0.862. Besides, precision and recall, which are commonly used for medical image classification, are appearing with the similar trend to AUC and ACC. As more intuitively illustrated in Figure 6, three CNN architectures combined with two datasets have produced six ROC Figures. The red curve denotes the two-stage scheme using tissue-wise “medium-level” dataset, while the green curve denotes the conventional one-stage scheme. It is clear at a glance, in each figure, that our proposed scheme possesses overwhelming area all along both the false-positive rate axis and true-positive rate axis. These results have proved that our proposed scheme is adaptable and rarely dependent on the amount of well-annotated data. In addition, a set of filtered response images exported by the stepwise trained network are displayed in Figure 7. Considering the practical and intuitive facility, we investigate on VGG-16 model and obtain some of the outcomes of the first convolutional layers from all of the convolutional blocks as shown.

In line with the common sense, larger training data yield larger absolute AUC value. Nevertheless, if we make comparison between the data size crosswise, we can observe that when we use the proposed two-stage scheme with the small dataset, our scheme has actually boosted the performance up to the level approaching to that when only one-stage fine-tuning is implemented with the large dataset which is 10 times that of the small one. By viewing the two rows “Small-Two stage (tissue)”and “Large-One stage” (right the two rows in the middle of Table 2), it is not hard to see that the differences of AUC values between the two rows are no larger than 0.023 (AlexNet), while the largest difference of ACC values is only 0.033 (AlexNet). These results can fully prove that the introduction of tissue-wise information has indeed contributed to making the deep neural networks “understand” pathology images better. Hence, it is reasoned to infer that our proposed two-stage fine-tuning scheme can be used as an alternative method to boost the performance when the number of well-annotated pathology image data is limited, but “nonprofessional” annotation is practicable in some degree.

### 4.2. Stepwise Fine-Tuning Using Cell-Wise Data

**Figure 8:**
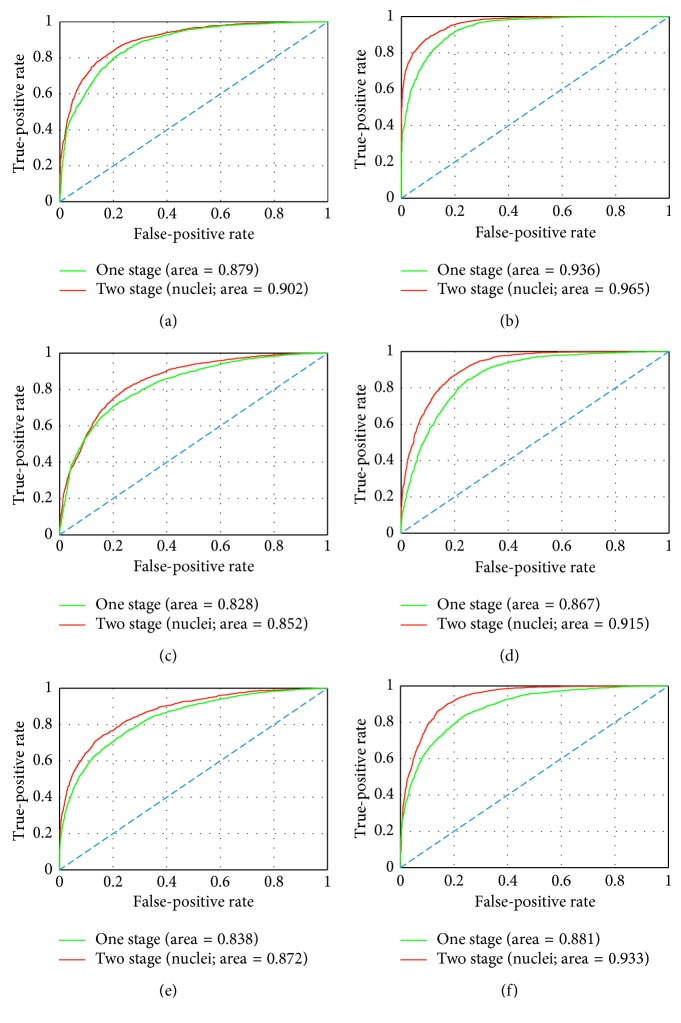
Performances of the proposed two-stage fine-tuning using cell-wise data presented by ROC. (a) VGG-16 + small data. (b) VGG-16 + big data. (c) AlexNet + small data. (d) AlexNet + big data. (e) Inception V3 + small data. (f) Inception V3 + big data.

In the next step, we will follow the same procedures as above but substitute the cell-wise data which have been generated by our proposed automatic clustering approach for the tissue-wise data. To make a comprehensive comparison of the rival schemes, again in Table 3, we list up the results of the two schemes covering two sizes of well-annotated datasets and three types of CNN architectures. From Table 3, we can find that the proposed stepwise fine-tuning scheme using cell-wise (nuclei) datasets as well governs all couples of results. According to the AUC values, when small well-annotated dataset is employed, the fine-tuning by nuclei datasets has led to improvement by 0.023 (VGG-16), 0.024 (AlexNet), and 0.034 (Inception V3), respectively, along the horizontal direction. In the large data group, AUC values for the three networks are separately elevated by 0.029 (VGG-16), 0.048 (AlexNet), and 0.052 (Inception V3). In agreement with AUC values, ACC values have been largely raised after we introduce the nuclei dataset into the first fine-tuning stage, with whichever CNN architecture and well-annotated dataset. As to the indexes precision and recall, in each couple of the small data group, our proposed scheme yields enhancement for both precision and recall. However, the results depicted in the large dataset group show decline of precision, but on the contrary, substantial increase of recall as offset. In line with the definitions, a larger recall indicates less undetected anomaly (false negative), while a larger prevision indicates less overdetection (false positive). Practically, due to the high risk of undetected anomaly, a high recall is acceptable rather than a loss of it. In Figure 8, the ROC performances of the two-stage fine-tuning using nuclei “medium-level” dataset are presented. Likewise, we notice that our proposed two-stage stepwise fine-tuning scheme oversteps the state-of-the-art one-stage fine-tuning approach regardless of the scale of “high-level” dataset. By implementing the two-stage fine-tuning scheme, the performances of the state-of-the-art deep neural networks are all boosted up to a competitive level close to those achieved by the usual one-stage fine-tuning with a much larger well-annotated “high-level” dataset.

Moreover, if we compare with the two types of “medium-level” datasets utilized in the first-stage fine-tuning, there are actually hardly apparent differences observed between them. When we have a small well-annotated dataset, tissue-wise data seem to promote more for VGG-16 and Inception V3 models, while the AlexNet seems to profit more from the cell-wise data. Correspondingly, when we have a large well-annotated dataset, AlexNet and Inception V3 are both reinforced more effectively. Therefore, to sum up, all of these results have demonstrated that the proposed stepwise fine-tuning employing either of the tissue-wise dataset or the cell-wise dataset has successfully boosted the performance of the pretrained neural networks for gastric pathology image classification in various situations.

## 5. Conclusions

In this paper, aiming to alleviate the insufficient well-annotated training data for pathology image classification, we proposed a novel stepwise fine-tuning scheme and a kind of low-cost target-correlative intermediate data with which the deep neural networks are supposed able to acquire fundamental pathological knowledge in accordance with pathologist's perception manner. To match the demand of different data production capabilities, we proposed two practical approaches to build up the intermediate data. Both of the two approaches incur no additional pathologist's workload. In the experiments, our proposed scheme exerted adequate efficacy for boosting the classification performance in respect of different possession situations of well-annotated pathology image data, and revealed high applicability for different state-of-the-art CNN architectures.

Last but not least, in this paper we regarded the final classification performance as the only assessment standard. To be objective, the effectiveness of the target-correlative intermediate data applied to the deep neural networks is expected to be concretely evaluated in the future work. Moreover, there is still necessity to discuss about the difference between the training framework using fine-tuning and other promising techniques such as multitask learning. Taking the proposed scheme as seed, we are looking forward to seeing that such kind of training scheme using low-cost target-correlative data can suggest solutions for more medical image classification tasks.

## Figures and Tables

**Table 1:** Datasets used in experiments.

Data type	Category	Training	Validation	Test
Tissue-wise data	Background	15,000	1,000	—
	Epithelium	15,000	1,000	—
	Stroma	15,000	1,000	—

Cell-wise (nuclei) data	Cluster 1	6,905	767	—
	Cluster 2	5,811	646	—

Well-augmented data	Small	540 + 540	1620 + 1620	2,700 + 2,700
	Large	5400 + 5400		

**Table 2:** Performances of the proposed two-stage fine-tuning using tissue-wise data.

Data size	Scheme	CNN architecture											
		VGG-16				AlexNet				GoogLeNet (Inception V3)			
		AUC	ACC	Precision	Recall	AUC	ACC	Precision	Recall	AUC	ACC	Precision	Recall
Small	One stage	0.879	0.793	0.863	0.695	0.828	0.723	0.74	0.72	0.838	0.753	0.75	0.75
	Two stage (tissue)	0.914	0.829	0.865	0.781	0.844	0.761	0.76	0.76	0.877	0.772	0.79	0.77

Large	One stage	0.936	0.836	0.957	0.703	0.867	0.794	0.80	0.79	0.881	0.779	0.79	0.78
	Two stage (tissue)	0.963	0.881	0.869	0.898	0.920	0.837	0.84	0.84	0.934	0.862	0.86	0.86

**Table 3:** Performances of the proposed two-stage fine-tuning using cell-wise (nuclei) data.

Data size	Scheme	CNN architecture											
		VGG-16				AlexNet				GoogLeNet (Inception V3)			
		AUC	ACC	Precision	Recall	AUC	ACC	Precision	Recall	AUC	ACC	Precision	Recall
Small	One stage	0.879	0.793	0.863	0.695	0.828	0.723	0.74	0.72	0.838	0.753	0.75	0.75
	Two stage (nuclei)	0.902	0.815	0.873	0.730	0.852	0.777	0.78	0.78	0.872	0.784	0.78	0.78

Large	One stage	0.936	0.836	0.957	0.703	0.867	0.794	0.80	0.79	0.881	0.779	0.79	0.78
	Two stage (nuclei)	0.965	0.89	0.881	0.901	0.915	0.834	0.84	0.83	0.933	0.862	0.86	0.86

## Data Availability

The data used in this paper were provided by Toho University Sakura Medical Center and are now currently managed by the National Institute of Advanced Industrial Science and Technology (AIST). However, as to the agreement on data usage, Toho University Sakura Medical Center only gained consent from the patients “The anonymized image data are provided to AIST and can be used for image analysis experiments”, which indicates only the right “to use.” Meanwhile, at Toho University Sakura Medical Center, a research plan to provide data only to AIST was approved, so it is not permissible to provide data from AIST to the outside under the existing agreement framework. In order to provide data to a third-party organization in any way, after acquiring consent from the patients and preparing an experiment plan specifying the provision of providing data to the outside, it is necessary to obtain the approval of the Ethics Review Committee of both institutions. Since it is difficult to carry out all of these procedures this time, unfortunately, we cannot make the data publicly available in the prescribed form.
